# Culture-Negative Fibrinous Peritonitis in a Postpartum Female

**DOI:** 10.7759/cureus.43339

**Published:** 2023-08-11

**Authors:** Anand Dhaliwal, Daniel I Razick, Nancy Le, Muzammil Akhtar, Joelle Jakobsen

**Affiliations:** 1 Surgery, California Northstate University College of Medicine, Elk Grove, USA; 2 Neurology, California Northstate University College of Medicine, Elk Grove, USA; 3 General Surgery, Mercy Medical Group, Sacramento, USA

**Keywords:** culture negative, laparatomy, postpartum, non-traumatic peritonitis, peritonitis

## Abstract

Peritonitis is inflammation of the peritoneum that can arise from a number of complications affecting the lining of the abdominal wall and organs. Acute abdomen and peritonitis is a rare complication in a previously healthy woman following a seemingly uncomplicated normal full-term vaginal delivery. We report such a case in a 20-year-old gravida 2 para 2 (G2P2) woman of Guatemalan descent, who presented nine days postpartum following an uncomplicated delivery, to the emergency room with acute abdomen and associated systemic inflammatory reaction. Interventional radiology paracentesis was performed, yielding a milky, purulent peritoneal fluid with no visible organisms and negative cultures. Antibiotics and paracentesis were insufficient in managing her condition, which continued to worsen over the course of several days. Given her continued deterioration despite clinical intervention, she underwent an exploratory laparotomy and peritoneal lavage along with continued broad-spectrum antibiotics. Cultures continued to be negative but operative findings included diffuse fibrinous peritonitis with no obvious abscess or perforated abdominal viscus. Following surgical laparotomy, she recovered fully without any complications. We review the available literature regarding peritonitis, discuss its management, and speculate as to its cause in this case.

## Introduction

Peritonitis is a frequently encountered emergent condition characterized by inflammation of the peritoneum [1]. It can be further divided into the two most commonly identified causes: spontaneous bacterial peritonitis (SBP) or secondary peritonitis. SBP is an ascitic fluid infection with no evidence of an intra-abdominal surgically treatable source [2]. Alternatively, secondary peritonitis is defined as an ascitic fluid infection with a positive bacterial culture and an ascitic fluid polymorphonuclear cell count over 250 cells/mm^3^, with evidence of a surgically treatable intra-abdominal infection source. The distinction is often made based on fluid analysis, imaging, and response to treatment [3]. 

Peritonitis classically presents with severe abdominal pain, abdominal tenderness and rigidity, fever, chills, and altered mental status [4]. If suspected, paracentesis should be conducted promptly as delaying this has been associated with increased mortality [5]. Since the majority of cases are caused by bacterial infections, spontaneous or secondary, empiric broad-spectrum antibiotic therapy is typically initiated. If antibiotic treatment is unsuccessful, exploratory laparotomy and peritoneal lavage is considered the definitive treatment to identify and clear the source of infection [6]. We present a rare case of postpartum peritonitis in a previously healthy 20-year-old patient, in which exploratory laparotomy with peritoneal lavage was required. 

## Case presentation

A 20-year-old gravida 2 para 2 (G2P2) Guatemalan female, with no past medical history, presented to the emergency department (ED) nine days following uncomplicated full-term pregnancy via vaginal delivery. She presented with a chief complaint of a three-day history of multiple episodes of nausea, vomiting, and abdominal cramping. The patient also stated over the last few days she had profuse, non-bloody dark diarrhea up to eight times per day, along with residual non-bloody vaginal discharge. Lower abdominal pain was rated as eight out of 10. She denied any recent sick contacts or travel. The patient reported taking acetaminophen and ibuprofen at home with minimal relief. 

Upon arrival, the patient was tachycardic with a heart rate of 124, respiratory rate of 28, blood pressure of 135/74, and temperature of 39.4 Celsius (102.9 F). Code sepsis was called with fluids, ceftriaxone, and acetaminophen. Physical examination revealed a soft and tender abdomen in all four quadrants on deep palpation, with mild guarding and suprapubic tenderness on palpation. Foul-smelling material was noted in undergarments, suggesting possible infectious colitis. She was promptly started on antibiotic treatment consisting of ceftriaxone, ampicillin, clindamycin, and gentamicin, as well as ondansetron, dicyclomine, and morphine. Electrocardiogram (EKG) showed sinus tachycardia with a rate of 120 but was otherwise normal. Complete blood count (CBC) and comprehensive metabolic panel (CMP) results revealed decreases in hemoglobin, hematocrit, sodium, and potassium, with elevated platelet count, bands, blood urea nitrogen (BUN), and glucose (Table 1).

**Table 1:** CBC and CMP results and reference ranges CBC, complete blood count; CMP, comprehensive metabolic panel; BUN, blood urea nitrogen.

Result name	Value	Reference range (female)
Hemoglobin (g/dL)	10.5	12.1-15.1
Hematocrit (%)	32.7	36-44
Sodium (mmol/L)	133	136-145
Potassium (mEq/L)	3.1	3.5-5.2
Platelets (/mcL)	457,000	150,000-400,000
Bands (%)	14	3-5
BUN (mg/dL)	37	7-20
Fasted glucose (mg/dL)	106	70-100

**Figure 1:**
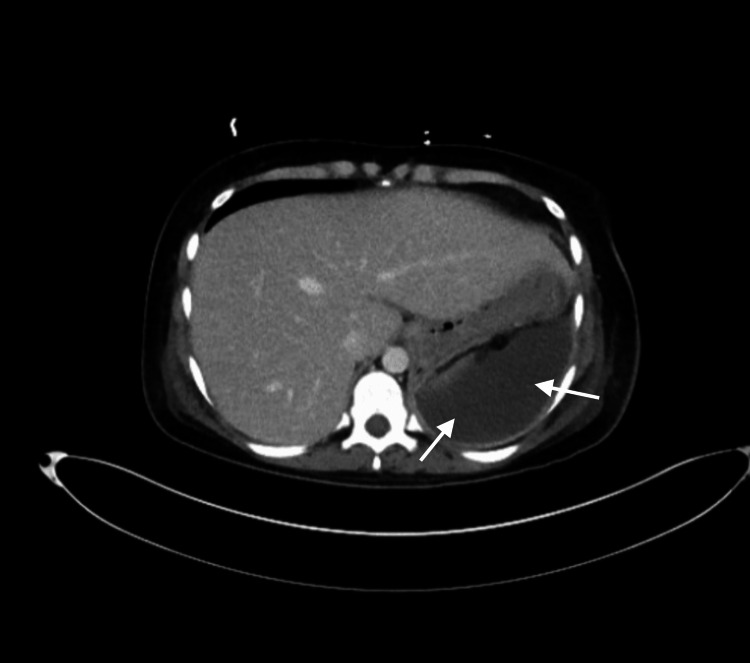
Transverse CT image of the abdomen prior to laparotomy showing perisplenic fluid collection CT, computed tomography.

Computed tomography (CT) of the abdomen and pelvis with intravenous contrast revealed an enlarged post-gravid uterus with air in the lower uterine segment of the endometrial cavity. Mild wall thickening diffusely throughout the entire large bowel was noted and was thought to possibly reflect infectious or inflammatory colitis. A large fluid collection superior to the spleen was also noted (Figure 1).

The patient continued to be persistently tachycardic and later developed fever. At this point, the concern was for endometritis with concurrent colitis after a vaginal exam. The patient was admitted with continued IV antibiotics and observation. 

**Figure 2:**
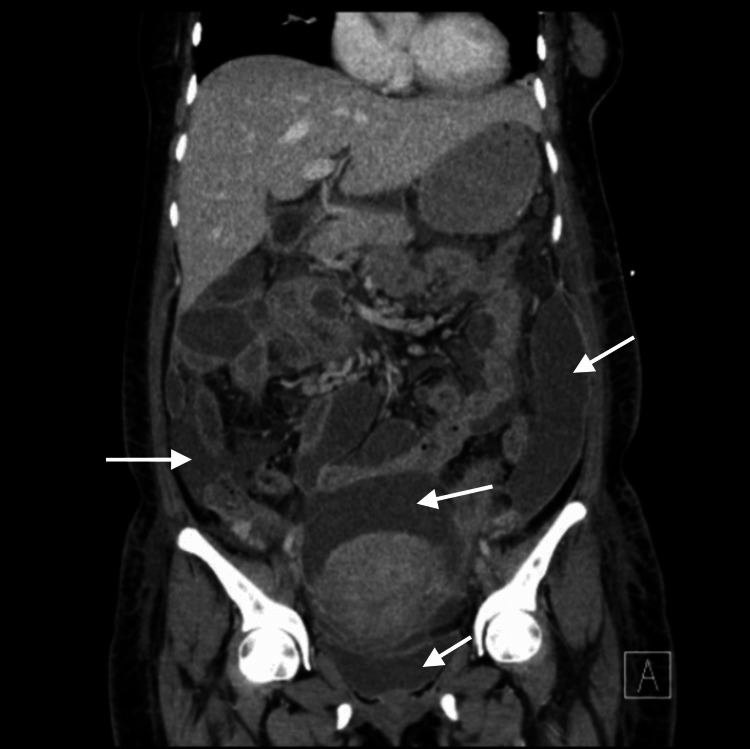
Coronal CT image of the abdomen/pelvis showing multiple fluid collections in the paracolic gutters and surrounding the uterus CT, computed tomography.

The patient remained admitted for three days and after her condition did not improve, antibiotics were changed to ceftriaxone and metronidazole. Laparotomy was planned as the patient’s condition continued to deteriorate. Interventional radiology paracentesis was performed yielding a minty, milky drainage. The fluid was cultured, though no bacteria growth was noted. Four days later, a repeat CT was done prior to the planned laparotomy, which showed multiple fluid collections of increased size and metabolic activity with remote enhancement relative to the first presentation, consistent with peritonitis (Figure 2). 

**Figure 3:**
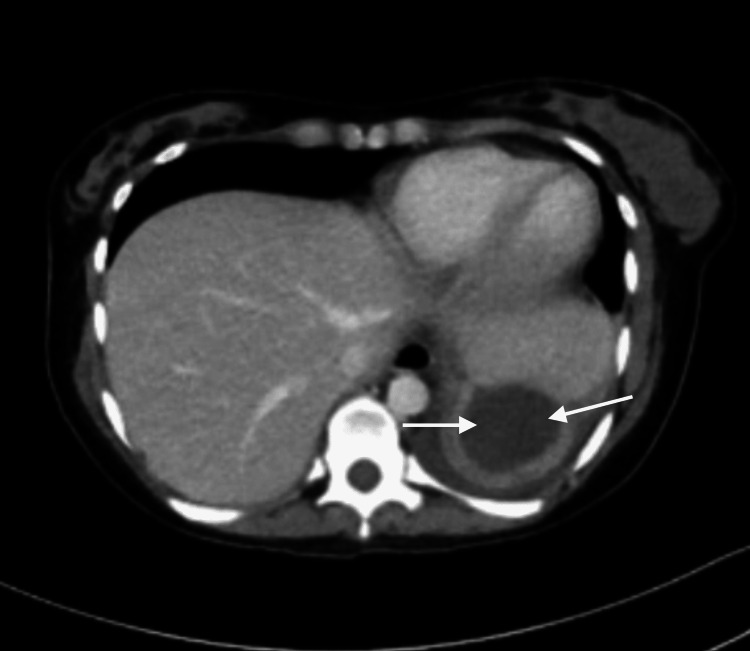
Transverse CT image of the abdomen one week postoperatively demonstrating decreased perisplenic fluid volume CT, computed tomography.

On admission day 8, the patient underwent an exploratory laparotomy with peritoneal lavage to clear the exudate and possible sources of inflammation. Operative findings included diffuse fibrinous peritonitis with no obvious perforation of abdominal viscus. Postoperatively, cultures continued to return negative for bacterial growth, possibly due to antibiotic treatment. Repeat CT was performed one week postoperatively and showed a small, partially loculated collection of fluid superior to the spleen, though a significantly decreased volume since the previous CT (Figure 3). 

The patient remained admitted to the hospital for three weeks and was discharged after making a complete recovery. She was prescribed docusate 100 mg twice per day for constipation relief. 

## Discussion

Peritonitis is typically categorized as spontaneous bacterial or secondary bacterial, though culture-negative peritonitis is extremely uncommon. While our patient presented with several of the classic symptoms such as severe abdominal pain, abdominal tenderness, and fever, repeated cultures of ascitic fluid yielded no bacterial growth over the course of her nearly five-week admission. A wide variety of antibiotic therapies were given to treat the condition and the patient was started on antibiotics immediately upon arrival at the ED, which could potentially explain the continued negative cultures. However, it was not until an exploratory laparotomy and peritoneal lavage were executed that the patient’s condition improved. Exploratory laparotomy coupled with peritoneal lavage is typically indicated in patients with penetrating abdominal wounds, where the source of bleeding cannot be identified with imaging [7]. This patient was unique in that there was no recent abdominal trauma or necrosis, she had no past medical history whatsoever, and her recent pregnancy and delivery were uncomplicated. 

The treatment of peritonitis in this patient was unique in that antibiotic treatment was unsuccessful. However, antibiotic therapy is indicated for patients who have any of the following: temperature above 37.8 degrees Celsius (100 degrees Fahrenheit), abdominal pain and/or tenderness, altered mental status, or ascitic fluid polymorphonuclear leukocyte count over 250 cells/microL. With regard to SBP in particular, third-generation cephalosporins such as cefotaxime are the preferred initial antibiotic [8]. Carbapenems are the second-line broad-spectrum class of choice, though they are typically reserved for patients in critical conditions or with severe disease. Recommended antibiotic treatment duration is five days, though this can be extended if the patient's condition does not improve [9]. Given the patient’s progressively worsening condition, a combination of ceftriaxone, metronidazole, ampicillin, azithromycin, clindamycin, doxycycline, gentamicin, and vancomycin was trialed with no success.

With regard to secondary bacterial peritonitis or SBP, distinguishing between the two is vital. The mortality of secondary bacterial peritonitis is nearly 100% if treatment consists of only antibiotics [3]. The mortality of SBP is nearly 80% if the patient undergoes exploratory laparotomy [10]. Therefore, it can be reasoned that most patients whose conditions do not improve with antibiotic therapy should undergo an exploratory laparotomy to identify and resolve the source of inflammation. 

Vernix caseosa peritonitis (VCP) is a rare complication post-cesarean section, with only 18 cases reported in the literature to date, characterized by acute abdomen days to weeks after delivery [11]. Vernix caseosa is a white, cheese-like skin covering unique to human newborns that acts as a skin cleanser [12,13]. While the exact pathophysiology of VCP is not fully understood, it is thought that VCP is a granulomatous reaction from the spillage of amniotic fluid or meconium into the maternal peritoneum [11]. We speculate VCP as a possible cause of this patient’s fibrinous peritonitis, though unlikely given the patient completed her delivery vaginally.

This case report has its limitations. Given the unique nature of this presentation, generalizing the findings of the report is difficult. We advise medical practitioners to consider peritonitis as a differential diagnosis despite cultures returning negative and recommend exploratory laparotomy with peritoneal lavage if antibiotic treatment proves unsuccessful.

## Conclusions

Although the patient’s extensive workup yielded no source of infection, surgical laparotomy with peritoneal lavage was beneficial in treating the acute abdomen. We speculate as to the causes of this patient’s fibrinous peritonitis, such as ante- or intrapartum VCP via uterine-tubal reflux; however, further studies of the patient’s laboratory specimens are warranted for diagnosis. The patient’s atypical culture-negative peritonitis with the associated systemic inflammatory response in the absence of trauma or necrosis is a unique presentation of a common clinical diagnosis. Peritonitis should remain on differential with a patient presenting with acute abdominal pain with fever and nausea/vomiting despite no past medical history or recent abdominal trauma. 
